# Abdominal Tuberculosis and Milk

**Published:** 1905-07-15

**Authors:** 


### ABDOMINAL TUBERCULOSIS AND MILK.

In a recent address by Professor Adami [1] attention is drawn to the statistics published by Kitasato dealing with tuberculosis in Japan. In these it is shown that the cases of fatal tuberculosis in Japan have just about the same proportion to the total deaths and to the population as is the case in European countries. The deaths from intestinal tuberculosis in persons under 18 years of age form rather a higher percentage than has been observed in Europe and America. The importance of these figures in relation to the opinion that intestinal tuberculosis depends upon infection from cow's milk is obvious; for such a method of feeding is unknown in Japan. There, if a mother is unable to feed her infant, a foster-mother is employed. Indeed very little milk is consumed in Japan. Calculations give an estimate that the individual Jap does not use more on an average than three-quarters of a teaspoonful daily. Further, Kitasato shows that bovine tuberculosis is unknown among native Japanese cattle. These observations go to support the view that a large proportion of the cases of European intestinal tuberculosis are in all probability not due to infection from milk, and that undue stress has been laid upon bovine tuberculosis as a cause of tubercle in human beings. There can be no doubt that professional opinion is tending more and more in this direction. In a recent discussion at one of the London societies several physicians threw doubt on the more commonly received opinion. Amongst them Dr. Walter Carr drew attention to the fact that abdominal tuberculosis was practically an unknown disease during the first year of life, whereas it is common at a later date when little milk is given. He suggested that the crawling habit might be a factor in determining infection. Dr. Garrod also emphasised the enormous preponderance of thoracic over abdominal tuberculosis in young infants as showing that the infrequency of the latter condition could not be due to an insusceptibility to the disease in very early life.

[1] Brit. Med. Jour., May 27, 1905.

